# National Institutes of Health Funding in Obstetrics and Gynecology: Analysis of R01 Grants by Degree and Gender

**DOI:** 10.7759/cureus.8170

**Published:** 2020-05-17

**Authors:** Erich J Berg, John Ashurst

**Affiliations:** 1 Medicine, Arizona College of Osteopathic Medicine, Phoenix, USA; 2 Emergency Medicine, Kingman Regional Medical Center, Kingman, USA

**Keywords:** osteopathic, obgyn, r01

## Abstract

Introduction: Limited data currently exist regarding the demographics of principal investigators (PIs) in obstetrics and gynecology (OBGYN), who have received an R01 grant from the National Institutes of Health (NIH).

Objective: This study investigated funding differences among gender and advanced degree for PIs in the disciplines of OBGYN.

Methods: Retrospective data were collected from the NIH Research Portfolio Online Reporting Tools Expenditures and Results (RePORTER) tool to identify OBGYN PIs who received an R01 grant between 2008 and 2017.

Results: Between 2008 and 2017, the NIH awarded 263 R01 grants totaling $113,326,883 in funding to investigators in OBGYN. Male PIs and PIs holding a non-medical degree were awarded the majority of R01 grants (52.47% and 55.51%, respectively). Zero osteopathic (Doctor of Osteopathic Medicine [DO]) physicians were awarded an R01 grant in OBGYN during this time period. Females were awarded larger dollar amounts than males ($449,556 vs $414,003, p=0.04). Allopathic (Doctor of Medicine [MD]) physicians were awarded larger grants than scientists holding a non-medical degree ($467,849 vs $401,291, p<0.01). Both male and female MD physicians were awarded more dollars per grant as compared with PIs holding a non-medical degree (p=0.01 and p<0.01, respectively).

Conclusions: Between 2008 and 2017, a degree disparity was found to exist for investigators who received an NIH R01 grant in OBGYN. Females and investigators holding an MD degree were awarded larger total grants than their male and non-physician counterparts. Further research needs to be undertaken to understand the degree disparity and recent funding trends by the NIH.

## Introduction

Research has been one of the driving forces of medical advancement and improving patient-centered healthcare outcomes over the last several decades. However, these advancements would not be possible without physician scientists and grant funding. The National Institutes of Health (NIH) has been the one of the largest funding sources for research over the last decade and the Research Project (R)-series category is a major funding source for new principal investigators (PIs) [1]. 

In 2018, departments in obstetrics and gynecology (OBGYN) represented only 1% of all total NIH funding despite being a major provider of primary care services to the population [2]. Although the exact cause of this disproportionate funding to departments of OBGYN is unknown, it could be related to funding trends within the NIH that directly award funds for women’s health. Only 4% of NIH R-series and 7% of the K-series career development grants were designated to the National Institute of Child Health and Human Development (NICHD) funding category that directly funds women’s health in 2018 [2]. 

Not only has the OBGYN specialty been awarded a small amount of grant funding over the years, but there has also been a decrease in the number of physician scientists in the community. Between 1985 and 2003, there was a decrease of 39.13% in the number of physicians who engaged in research as their primary professional activity [3]. Recent trends have also emerged that a degree disparity exists in those physician scientists who not only publish in high-ranking journals but also those who receive NIH R01 grant funding [4-8]. In this study, the authors sought to determine recent trends in NIH R01 grant funding in OBGYN between 2008 and 2017. 

## Materials and methods

Following a similar methodology as Berg and Ashurst, an advanced search was performed using the NIH Research Portfolio Online Reporting Tools Expenditures and Results (RePORTER) search engine (http://projectreporter.nih.gov) for the “obstetrics and gynecology” keyword and the R01 activity code for the fiscal years between 2008 and 2017 [4,5]. Demographic data were collected for each PI who received an R01 award and included gender, total dollar amount awarded, medical degree (osteopathic or allopathic), dual degree (a combination of a medical degree and non-medical degree), and other degrees (PhD, DSc, MPH, etc.) obtained. PIs were not excluded if awarded more than one grant over the study period, and each grant awarded was tallied. Only new R01 grants were recorded for each fiscal year, and the total grant amount was rounded to the nearest dollar. 

Comparisons of the proportions of gender and degree(s) from each year were determined by using descriptive statistics, and comparisons of each group were completed by a one-tailed t-test. Statistical significance was defined as p≤0.05. 

## Results

Over the decade studied, a total of 263 R01 grants were awarded to OBGYN departments and had a total net worth of $113,326,883. Of the grants awarded, 52.47% (138/263) were to the male gender and 44.49% (117/263) were awarded to allopathic physician scientists (Doctor of Medicine [MD]) (Table 1). No osteopathic physician scientist (Doctor of Osteopathic Medicine [DO]) was awarded a R01 grant in OBGYN over the time studied. On average, females were awarded larger grants based upon dollar amount as compared to their male counterparts ($449,556 vs $414,003; p=0.04). Those PIs who held a medical degree were also awarded larger grants based upon total dollar amount as compared to those holding a degree designated as other ($467,849 vs $401,291; p<0.01). A total of 58.12% of MDs held a dual degree and no statistically significant difference was noted in the total dollar amount of grants awarded to those MDs holding a dual degree versus those holding only a degree in medicine ($459,762 vs $479,475; p=0.30).

**Table 1:** Percentage of male and female with MDs, DOs, and those holding a degree designated as "other" receiving NIH R01 grants in OBGYN from 2008 to 2017 DO: Doctor of Osteopathic Medicine, MD: Doctor of Medicine, NIH: National Institutes of Health, OBGYN: Obstetrics and Gynecology

Gender	Degree Designation		
	MD	DO	Other
Male	42.75% (59/138)	0% (0/138)	57.25% (79/138)
Female	45.60% (57/125)	0% (0/125)	54.40% (68/125)

When gender and degree were considered, the degree designated as "other" comprised 54.40% of all grants awarded to the female gender, but those females with a MD degree were awarded grants with a larger total dollar amount ($485,125 vs $419,741; p=0.01) (Table 2).

**Table 2:** Average NIH R01 dollar amount awarded to the different genders and degrees in OBGYN from 2008 to 2017 MD: Doctor of Medicine, NIH: National Institutes of Health,OBGYN: Obstetrics and Gynecology

Gender	Degree Designation		
	MD	Other Degree	P-Value
Male	451,1437	385,206	<0.01
Female	485,125	419,741	0.01

A total of 57.90% of all female MDs held dual degrees, but no statistically significant difference was noted in the total dollar amount awarded per grant between those female MDs holding a dual degree and those with only a medical degree ($496,374 vs $477,411; p=0.31) (Table 3). 

**Table 3:** Average OBGYN NIH R01 grants in dollar amount awarded to allopathic physicians with an MD and allopathic physicians holding a dual degree based on gender MD: Doctor of Medicine, NIH: National Institutes of Health, OBGYN: Obstetrics and Gynecology

Gender	Degree Designation		
	MD	Dual Degree	P-Value
Male	426,202	489,291	0.11
Female	496,374	477,411	0.31

When males were considered, 57.25% held a degree designated as "other," but MDs were awarded larger grants based upon total dollar value ($451,1437 vs $385,206; p<0.01) (Table 2). A total of 61.02% of males with a MD degree held dual degrees, but no statistically significant difference was noted between MD males with dual degrees and those with only a medical degree when the total dollar amount of grant awarded was considered ($426,202 vs $489,291; p=0.11) (Table 3). 

## Discussion

The number of physician scientists has decreased over the last decade despite an increase in the overall physician workforce [9]. Based upon the current data, the majority of NIH R01 grants in OBGYN over the time period studied were awarded to those who did not hold a medical degree. This degree disparity could be related to the duration of residency and fellowship training, medical school debt, concerns around work-life balance, clinical service demands after the completion of training, and the overall lack of academic mentorship for those in current OBGYN practice [9]. 

When comparing the gender of those who received a R01 grant in OBGYN, males and females are almost equally represented. The near gender equality in those receiving an NIH R01 grant could be related to an increase in both female faculty members and residents in OBGYN over the last several years [9]. Recent data have also shown that females are now the majority of K grant recipients in OBGYN which could have led to increased mentorship for those applying for an R01 grant [9]. 

No osteopathic physician received an NIH R01 grant in OBGYN over the time period studied. Similar to these results, no osteopathic physician has been awarded an NIH R01 grant in family medicine, general surgery, and emergency medicine over a similar time period [4-6]. It is unclear the reasoning behind the lack of NIH R01 grant funding in osteopathic OBGYNs, but it could be related to the lack of original research publications by osteopathic physicians in high-ranking OBGYN journals [7]. Without a prior track record in scholarly activity, it could be difficult for an osteopathic physician in OBYGN to receive an NIH R01 grant. The lack of mentorship within the osteopathic community for research could also contribute to these findings [10]. 

Typically, those who hold a dual degree in medicine and another academic field undergo significant research training prior to graduation. In general surgery, males with a dual degree have been awarded larger R01 grant funds as compared to those with only a medical degree [4]. However, there was no difference in OBGYN R01 grant funding in those males and females who held either a dual degree or a degree in medicine only over the last decade. The cause of these results is unclear, but it could be related to recent data showing that having a dual degree does not serve as a barrier to obtaining research funding [9]. 

## Conclusions

Over the decade studied, a degree disparity was found to exist in those receiving an NIH R01 grant in OBGYN. Females and those holding an MD degree were awarded larger total grants than their counterparts, but no difference was seen for those MDs holding a dual degree. Further research needs to be undertaken to understand the degree disparity and recent funding trends by the NIH in the specialty of OBGYN. 
